# Save Your Strength

**Published:** 1889-08

**Authors:** 


### SAVE YOUR STRENGTH.

Young mothers, be as chary of your strength as a miser of his money. You will have abundant use for all at your command, in the rearing of your children. All used unnecessarily is wasted, squandered. You have a certain life supply, and when this is exhansted you must fail, though that exhaustion may occur at forty years of age. Like the moments, never returning, the vital supply, that was intended for the whole life, cannot return when once wasted. Let little feet run up and down stairs, to do little errands ; it will do the children no harm to do that part, and will favor you very much. Do not lift a whole tub, or even a pail of water, if it in any way over exerts you. A little planning, a little time taken for a hard effort, a little rest taken when you are weary, will prove economy. Overwork is as disastrous as the payment of exorbitant interest.

